# Reducing lower leg amputations in diabetes: a challenge for patients, healthcare providers and the healthcare system

**DOI:** 10.1007/s00125-012-2588-z

**Published:** 2012-05-24

**Authors:** N. C. Schaper, J. Apelqvist, K. Bakker

**Affiliations:** 1 Division of Endocrinology, Department of Internal Medicine, CAPHRI and CARIM Research Institutes, Maastricht University Medical Center+, PO Box 5800, 6202 Maastricht, the Netherlands; 2 Department of Endocrinology, University Hospital of Malmö, Malmö, Sweden; 3 International Working Group on the Diabetic Foot, Heemstede, the Netherlands

**Keywords:** Amputation, Diabetes, Diabetic foot, Multidisciplinary foot team, Peripheral arterial disease, Ulcer

## Abstract

Amputation of the lower limb is one of the most feared diabetic complications. It is associated with loss of mobility and a poor quality of life. Amputations result in high economic burden for the healthcare system. The financial cost is also high for patients and their families, particularly in countries that lack a comprehensive health service and/or have a low income. Losing a leg frequently implies financial ruin for a whole family in these countries; therefore, a reduction in diabetes-related amputations is a major global priority. Marked geographical variation in amputation rates has been reported within specific regions of an individual country and between countries. A coordinated healthcare system with a multidisciplinary approach is essential if the number of amputations is to be reduced. This commentary discusses how studies on the variation in amputation rates can help to identify barriers in the access or delivery of care with the aim of reducing the burden of diabetic foot disease.

In this issue of *Diabetologia* Holman and co-workers describe a worrisome variation in the recorded incidence of lower extremity amputations in England [1]. Based on data reported by all National Health Service (NHS) hospitals, the authors calculated the number of minor (below-ankle) and major (above-ankle) amputations per year in adults and combined this with data from other sources to estimate the amputation rate per 1,000 person-years in both diabetic and non-diabetic subjects. The incidence of total amputations (minor plus major) varied eightfold across Primary Care Trusts in both patients with diabetes (range, 0.64–5.25 per 1,000 person-years) and patients without diabetes (0.03–0.24 per 1,000 person-years). These important data are in line with several other studies that reported marked differences in amputation rate within specific regions of a country and between countries [2, 3].

Amputations are usually preceded by a foot ulcer and the most important factors predicting a poor outcome of these ulcers are the extent of tissue loss, infection, peripheral arterial disease (PAD) and co-morbidity [2–4]. The reasons for a major amputation are limited; the most frequent reasons are critical limb ischaemia with rest pain or progressive infection in a leg that cannot be successfully revascularised [5]. Sometimes an immediate amputation is performed because of life-threatening infection or infection with massive tissue loss. In addition, a minor amputation is frequently performed for a forefoot abscess, osteomyelitis or gangrene of a toe [5]. If other options are exhausted or undesirable, amputation can therefore be a treatment and not a failure.

As reviewed in 2004 in this journal, there are many factors that determine the amputation rate and pinpointing why it varies so markedly in England and elsewhere is a challenge [2] (Fig. 1). Part of the variability reported in the paper by Holman et al [1] could be explained by ethnic differences [6] but, owing to the study design, there is no information on disease severity or management. In the prospective European Study Group on Diabetes and the Lower Extremity (Eurodiale) study, which was performed in 1,232 diabetic patients with a new foot ulcer from all over Europe, the number of major amputations was too low (5%) to analyse, but the minor amputation rate varied markedly between the participating centres, from 2% to 33% [7]. As all patients underwent a comprehensive evaluation, a disease severity score could be calculated for each patient and a large part of the variation could be explained by differences in disease severity (*r* = 0.75). Amputation rate should therefore not be used as a quality indicator in diabetic foot disease, unless it can be corrected for the relevant characteristics of the patient, the leg and the foot.

**Fig. 1:**
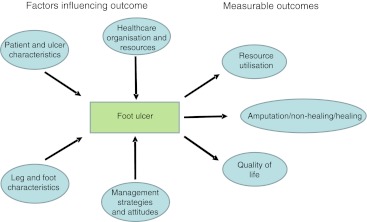
The outcome of diabetic foot ulcers is determined by patient and ulcer characteristics, by the local healthcare organisation and availability of resources, by the management strategies used and the attitudes of the care providers and patients. The outcome can be described in clinical terms, such as healing or amputation, in terms of quality of life or in terms of use of resources

The management of a diabetic foot ulcer requires a multidisciplinary approach, including revascularisation and surgical procedures as well as treatment of infection, oedema, pain, metabolic disturbances, malnutrition, co-morbidities, meticulous wound care and biomechanical offloading [5]. A coordinated system of support for both patients and carers is necessary to successfully implement these strategies, and if such an integrated approach is used, 45–85% of all amputations can be avoided [8–10]. The creation of a national network of specialised diabetic foot teams that are accessible to every patient should be the first priority for the prevention of amputations. England is a country with a single nationalised healthcare system, therefore the wide geographical variation in amputation rate cannot be explained by differences in reimbursement, availability of resources or access to care but point to differences in the organisation or delivery of care, as suggested by Holman et al [1]. Treatment delay because of underestimation of disease severity, unawareness of the potential dangers of a foot ulcer, or not recognising that it is a sign of multi-organ disease, by both the patient and the doctor can lead to a poor outcome. Initial treatment by physicians is frequently empirical, involving dressings and antibiotics. Only when the ulcer does not heal or deteriorates are patients referred for systematic management [11]. Up to 50% of the patients with a diabetic foot ulcer have PAD and infected ischaemic ulcers in particular have a poor prognosis; spreading of infection can be extremely rapid and ‘time is tissue’ in these ulcers [5]. In one study, late referral was associated with larger ulcers that had a poorer prognosis [3]. In Sweden, the amputation rate was lower in a region where direct referral by district nurses to a specialised centre was possible compared with a region where patients could only be referred by the GP [11]. Moreover, in another study a structured foot care programme with early detection of ulcers for patients at a high-risk of foot ulcers resulted in a reduction in amputations [12]. A nationwide system for the early detection and subsequent referral to a specialised diabetic foot team should therefore be implemented to reduce the amputation rate. Such a specialised multidisciplinary foot team should preferably include a diabetologist, vascular surgeon, endovascular specialist, podiatrist, shoe technician and specialised nurse. Clear guidelines have been formulated as regards the identification of at-risk patients, diagnosis of a foot ulcer, classification of disease severity and management [5]. Several elements of these guidelines have subsequently been validated in prospective studies [13–16].

Interestingly, Holman et al [1] observed that amputation rates in adults with diabetes were correlated with amputation rates in adults without diabetes (*r* = 0.433), pointing to a common factor. Differences in PAD management might explain part of the observed geographical variation in both diabetic and non-diabetic patients. In an earlier study in England, there was marked variation between vascular surgeons in terms of the procedure chosen when given the choice of amputation or revascularisation in a series of clinical cases [17]. A recent systematic review reported that in diabetic patients with critical limb ischaemia who were not revascularised, the 1 year amputation rate was 57% compared with a rate of 15–20% in patients who were revascularised [18]. Moreover, new techniques and technologies have been introduced for treating PAD; in particular, endovascular approaches in the lower limb have produced promising results. If and to what extent differences in PAD management contribute to the geographical variation in amputation rate remains to be determined in future studies. However, each diabetic patient with a (neuro-)ischaemic foot ulcer should be treated by a multidisciplinary team that provides the most appropriate and up-to-date therapy, i.e. an open (surgical) and/or an endovascular revascularisation procedure.

Although amputation seems a clearly defined endpoint, there are several caveats in the study by Holman et al [1]; besides those discussed above, there are also methodological issues. In reporting amputation rate the following should be defined in future studies: which amputation in a sequence is used as the outcome measure (i.e. the first amputation, the number of amputations or the final amputation level), are the individuals or are the number of limbs undergoing amputation reported and are the results based on the number of hospitalisations. When calculations are based on hospital admissions, a hospital can be penalised for good clinical care. For example, an immediate minor amputation can be indicated in infected neuro-ischaemic ulcers, followed by a revascularisation procedure once infection is under control. Subsequently, the patient is treated at home as restoration of skin microcirculation can take weeks, and is then re-admitted for a final operation, which can include a second minor amputation, to produce a foot that remains functional. In the Holman et al study, two amputations as part of a planned revascularisation could be interpreted as a worse outcome than a single major amputation. Moreover, in most studies, amputations are lumped together. However, a single toe, a whole forefoot or an above-knee amputation all have a different impact on quality of life and, in future studies, amputation levels should be reported. Finally, Holman et al do not describe the indication, the immediate cause of amputation or disease severity; these key data should be included in future studies for interpretation of the data.

Documentation of the geographical variation in amputation rate within a healthcare system can help to identify barriers to the access and/or delivery of care that need to be removed to reduce the incidence of diabetes-related amputations. But, as discussed above, the amputation rate is not a good marker of the quality of care, and studies similar to that reported in the Holman et al paper should be used as a starting point in a process to improve the outcome of diabetic foot ulcers. There are four major decision points in the prevention of an amputation in a patient with a foot ulcer: early referral to a multidisciplinary team, aggressive (surgical and medical) management of infection, diagnosis of PAD with the appropriate tests and revascularisation in the case of impaired tissue perfusion. As shown in the Eurodiale study, even in centres with a specific interest in the diabetic foot, the standard of care was suboptimal for many patients, resulting from a lack of clear referral guidelines, financial barriers caused by inadequate reimbursement, a lack of availability of staff and the personal beliefs of the doctors [19]. Treating these ulcers is a challenge for patients, care givers and healthcare systems. Treatment should not be focused solely on ulcer healing; diabetic foot disease is a lifelong condition, which means that the patient is always at risk of a new ulcer, amputation or early death, and so a holistic approach is needed to management as well as prevention.

## Abbreviations

Eurodiale: European Study Group on Diabetes and the Lower Extremity
PAD: Peripheral arterial disease

## References

[1] Holman N, Young RJ, Jeffcoate WJ (2012) Variation in the recorded incidence of amputation of the lower limb in England. Diabetologia. doi:10.1007/s00125-012-2468-6 10.1007/s00125-012-2468-6 22398645

[2] Jeffcoate WJ, van Houtum WH (2004). Amputation as a marker of the quality of foot care in diabetes. Diabetologia.

[3] Prompers L, Schaper N, Apelqvist J (2008). Prediction of outcome in individuals with diabetic foot ulcers: focus on the differences between individuals with and without peripheral arterial disease. The EURODIALE Study. Diabetologia.

[4] Gershater MA, Löndahl M, Nyberg P (2009). Complexity of factors related to outcome of neuropathic and neuroischaemic/ischaemic diabetic foot ulcers: a cohort study. Diabetologia.

[5] Bakker K, Apelqvist J, Schaper NC (2012). Practical guidelines on the management and prevention of the diabetic foot 2011. Diab Metab Res Rev.

[6] Chaturvedi N, Abbott CA, Whalley A (2002). Risk of diabetes-related amputation in South Asians vs Europeans in the UK. Diabet Med.

[7] van Battum P, Schaper N, Prompers L (2011). Differences in minor amputation rate in diabetic foot disease throughout Europe are in part explained by differences in disease severity at presentation. Diabet Med.

[8] Fusilli D, Alviggi L, Seghieri G, de Bellis A (2007). Improvement of diabetic foot care after the implementation of the International Consensus on the Diabetic Foot (ICDF): Results of a 5-year prospective study. Diabetes Res Clin Pract.

[9] Krishnan S, Nash F, Baker N (2008). Reduction in diabetic amputations over 11 years in a defined UK population: benefits of multidisciplinary team work and continuous prospective audit. Diabetes Care.

[10] Canavan RJ, Unwin NC, Kelly WF, Connolly VM (2008). Diabetes and nondiabetes related lower extremity amputation incidence before and after the introduction of better organized diabetes foot care: continuous longitudinal monitoring using a standard method. Diabetes Care.

[11] Apelqvist J, Larsson J (2000). What is the most effective way to reduce incidence of amputation in the diabetic foot?. Diabetes Metab Res Rev.

[12] McCabe CJ, Stevenson RC, Dolan AM (1998). Evaluation of a diabetic foot screening and protection programme. Diab Med.

[13] Peters EJ, Lavery LA, International Working Group on the Diabetic Foot (2001). Effectiveness of the diabetic foot risk classification system of the International Working Group on the Diabetic Foot. Diabetes Care.

[14] Lavery LA, Armstrong DG, Murdoch DP, Peters EJ, Lipsky BA (2007). Validation of the Infectious Diseases Society of America’s diabetic foot infection classification system. Clin Infect Dis.

[15] Abbas ZG, Lutale JK, Game FL, Jeffcoate WJ (2008). Comparison of four systems of classification of diabetic foot ulcers in Tanzania. Diabet Med.

[16] Sotto A, Richard JL, Combescure C (2010). Beneficial effects of implementing guidelines on microbiology and costs of infected diabetic foot ulcers. Diabetologia.

[17] Connelly J, Airey M, Chell S (2001). Variation in clinical decision making is a partial explanation for geographical variation in lower extremity amputation rates. Br J Surg.

[18] Hinchliffe RJ, Andros G, Apelqvist J (2012). A systematic review of the effectiveness of revascularization of the ulcerated foot in patients with diabetes and peripheral arterial disease. Diabetes Metab Res Rev.

[19] Prompers L, Huijberts M, Apelqvist J (2008). Delivery of care to diabetic patients with foot ulcers in daily practice: results of the Eurodiale Study, a prospective cohort study. Diabet Med.

